# The venom-gland transcriptome of the eastern diamondback rattlesnake (*Crotalus adamanteus*)

**DOI:** 10.1186/1471-2164-13-312

**Published:** 2012-07-16

**Authors:** Darin R Rokyta, Alan R Lemmon, Mark J Margres, Karalyn Aronow

**Affiliations:** 1 Department of Biological Science, Florida State University, Tallahassee, FL 32306-4295, USA; 2 Department of Scientific Computing, Florida State University, Tallahassee, FL 32306-4120, USA

## Abstract

**Background:**

Snake venoms have significant impacts on human populations through the morbidity and mortality associated with snakebites and as sources of drugs, drug leads, and physiological research tools. Genes expressed by venom-gland tissue, including those encoding toxic proteins, have therefore been sequenced but only with relatively sparse coverage resulting from the low-throughput sequencing approaches available. High-throughput approaches based on 454 pyrosequencing have recently been applied to the study of snake venoms to give the most complete characterizations to date of the genes expressed in active venom glands, but such approaches are costly and still provide a far-from-complete characterization of the genes expressed during venom production.

**Results:**

We describe the *de novo* assembly and analysis of the venom-gland transcriptome of an eastern diamondback rattlesnake (*Crotalus adamanteus*) based on 95,643,958 pairs of quality-filtered, 100-base-pair Illumina reads. We identified 123 unique, full-length toxin-coding sequences, which cluster into 78 groups with less than 1% nucleotide divergence, and 2,879 unique, full-length nontoxin coding sequences. The toxin sequences accounted for 35.4% of the total reads, and the nontoxin sequences for an additional 27.5%. The most highly expressed toxin was a small myotoxin related to crotamine, which accounted for 5.9% of the total reads. Snake-venom metalloproteinases accounted for the highest percentage of reads mapping to a toxin class (24.4%), followed by C-type lectins (22.2%) and serine proteinases (20.0%). The most diverse toxin classes were the C-type lectins (21 clusters), the snake-venom metalloproteinases (16 clusters), and the serine proteinases (14 clusters). The high-abundance nontoxin transcripts were predominantly those involved in protein folding and translation, consistent with the protein-secretory function of the tissue.

**Conclusions:**

We have provided the most complete characterization of the genes expressed in an active snake venom gland to date, producing insights into snakebite pathology and guidance for snakebite treatment for the largest rattlesnake species and arguably the most dangerous snake native to the United States of America, *C. adamanteus*. We have more than doubled the number of sequenced toxins for this species and created extensive genomic resources for snakes based entirely on *de novo* assembly of Illumina sequence data.

## Background

Human envenomation by snakes is a worldwide issue that claims more than 100,000 lives per year and exacts untold costs in the form of pain, disfigurement, and loss of limbs or limb function [1-3]. Despite the significance of snakebites, their treatments have remained largely unchanged for decades. The only treatments currently available are traditional antivenoms derived from antisera of animals, usually horses [4], innoculated with whole venoms [5,6]; such an approach is the only readily available option for largely uncharacterized, complex mixtures of proteins such as snake venoms. Although often lifesaving and generally effective against systemic effects, these antivenoms have little or no effect on local hemorrhage or necrosis [7-9], which are major aspects of the pathology of viperid bites and can result in lifelong disability [4,5]. These traditional treatments also sometimes lead to adverse reactions in patients [6]. Advances in treatment approaches will depend on a complete knowledge of the nature of the offending toxins, but current estimates of the numbers of unique toxins present in snake venoms are in excess of 100 [10], a number not approached in even the most extensive venom-characterization efforts to date [11].

The significance of snake venoms extends well beyond the selective pressures they may directly impose upon human populations. Snake venoms have evolutionary consequences for those species that snakes prey upon [12,13], as well as species that prey upon the snakes [14], and their study can therefore provide insights into predator-prey coevolution. Snake venom components have been leveraged as drugs and drug leads [15-17] and have been used directly as tools for studying physiological processes such as pain reception [18]. In addition to the significance of the toxins, the nature of the extreme specialization of snake venom glands for the rapid but temporary production and export of large quantities of protein could provide insights into basic mechanisms of proteostasis, the breakdown of which is thought to contribute to neurodegenerative diseases such as Parkinson’s and Alzheimer’s [19].

The eastern diamondback rattlesnake (*Crotalus adamanteus*) is a pit viper native to the southeastern United States and is the largest member of the genus *Crotalus*, reaching lengths of up to 2.44 m [20]. The diet of *C. adamanteus* consists primarily of small mammals (e.g., squirrels, rabbits, and mouse and rat species) and birds, particularly ground-nesting species such as quail [20]. Because of its extreme size and consequent large venom yield, *C. adamanteus* is arguably the most dangerous snake species in the United States and is one of the major sources of snakebite mortality throughout its range [21]. *Crotalus adamanteus* has recently become of interest from a conservation standpoint because of its declining range, which at one time included seven states along the southeastern Coastal Plain [22]. This species has now apparently been extirpated from Louisiana and is listed as endangered in North Carolina [23,24]. As a consequence of recent work by Rokyta et al. [11] based on 454 pyrosequencing, the venom of *C. adamanteus* is among the best-characterized snake venoms; 40 toxins have been identified.

Transcriptomic characterizations of venom glands of snakes [25-28] and other animals [29-32] have relied almost exclusively on low-throughput sequencing approaches. Sanger sequencing, with its relatively long, high-quality reads, has been the only method available until recently and has provided invaluable data on the identities of venom genes. Because venomous species are primarily nonmodel organisms, high-throughput sequencing approaches have been slow to pervade the field of venomics (but see Hu et al. [33]), despite becoming commonplace in other transcriptomic-based fields. Rokyta et al. [11] recently used 454 pyrosequencing to characterize venom genes for *C. adamanteus*. More recently, Durban et al. [34] used 454 sequencing to study the venom-gland transcriptomes of a mix of RNA from eight species of Costa Rican snakes. Whittington et al. [30] used a hybrid approach with both 454 and Illumina sequencing to characterize the platypus venom-gland transcriptome, although they had a reference genome sequence, making *de novo* assembly unnecessary. Pyrosequencing is expensive and low-throughput relative to Illumina sequencing, and the high error rate, particularly for homopolymer errors [35], significantly increases the difficulty of identifying coding sequences without reference sequences.

We sequenced the venom-gland transcriptome of the eastern diamondback rattlesnake with Illumina technology using a paired-end approach coupled with short insert sizes effectively to produce longer, high-quality reads on the order of approximately 150 nt to facilitate *de novo* assembly (an approach similar to that of Rodrigue et al. [36] for metagenomics). The difference in read length from that of 454 sequencing was compensated for by the increase of more than two orders of magnitude in the number of reads. We demonstrated *de novo* assembly and analysis of a venom-gland transcriptome using only Illumina sequences and provided a comprehensive characterization of both the toxin and nontoxin genes expressed in an actively producing snake venom gland.

## Results and discussion

### Venom-gland transcriptome sequencing and assembly

**Figure 1:**
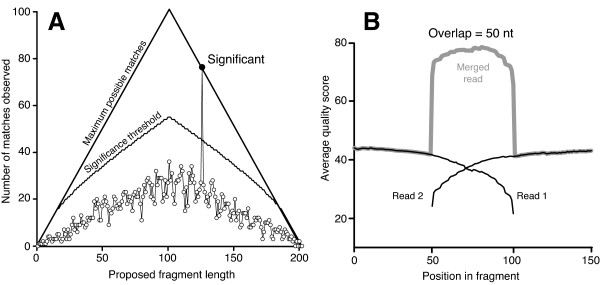
**Merging overlapping reads.** (**A**) Reads are slid along each other until the number of matches exceeds the significance threshold. In the example shown, the optimal overlap is 74 nucleotides (nt). (**B**) The quality of reads declines dramatically toward their 3’ ends, where overlap occurs if the fragment length is less than twice the read length, allowing the actual quality to be much higher than the nominal values. The example shown is the average of pairs that overlap by exactly 50 nt.

We generated a total of 95,643,958 pairs of reads that passed the Illumina quality filter for > 19 gigabases (Gb) of sequence from a cDNA library with an average insert size of ∼170 nt. Of these reads, 72,114,709 (75%) were merged (see Methods) on the basis of their 3’ overlap (Figure 1), yielding composite reads of average length 142 nt with average phred qualities > 40 and a total length > 10 Gb. This merging of reads reduced the effective size of the data set without loss of information and provided long reads to facilitate accurate assembly.

Our first approach to transcriptome assembly was aimed at identifying toxin genes. We attempted to use as many of the data as possible to ensure the identification of even the lowest-abundance toxins. To this end, we conducted extensive searches of assembly parameter space for both ABySS [37,38] (Table 1) and Velvet [39] on the basis of the full set of both merged and unmerged reads. We used the assemblies with the best *N*50 values for further analysis. For Velvet, the assembly using a *k*-mer size of 91 was best (*N*50=408); this assembly was subsequently analyzed with Oases [40]. For ABySS, the best *k*-mer value was also 91 (*N*50 = 2,007), but because the performance in terms of full-length transcripts appeared to depend strongly on the coverage (*c*) and erode (*e*) parameters, we further analyzed the *k* = 91 assemblies with *c* = 10 and *e* = 2, *c* = 100 and *e* = 100, and *c* = 1000 and *e* = 1000. We identified all full-length toxins by means of blastx searches on the results of all four assemblies.

**Table 1:** ABySS assembly summaries

			**Total**	**Longest**	**Contigs**	**Contigs**				**Total**
***k***	***c***	***e***	**contigs**	**contig**	**> 200 nt**	**>** *** N*** **50**	**Median**	**Mean**	***N*** **50**	**length**
51	2	2	2,168,050	15,079	147,942	27,410	364	592	790	8.77 × 10^7^
51	10	2	329,609	17,493	37,575	6,399	571	985	1,635	3.70 × 10^7^
51	10	10	337,039	17,459	37,944	6,390	554	967	1,621	3.67 × 10^7^
51	20	20	191,367	15,906	27,546	4,931	529	878	1,401	2.42 × 10^7^
51	30	30	135,961	13,472	21,986	4,034	494	812	1,256	1.79 × 10^7^
51	50	50	87,092	10,380	15,461	2,955	463	737	1,088	1.14 × 10^7^
51	100	10	42,366	8,553	8,510	1,725	432	658	906	5.60 × 10^6^
51	100	100	42,251	8,552	8,401	1,707	431	656	899	5.52 × 10^6^
51	1000	1000	2,319	5,232	571	123	428	631	797	3.61 × 10^5^
61	2	2	1,769,274	17,166	141,471	25,105	361	604	827	8.55 × 10^7^
61	10	2	263,688	17,493	34,076	5,959	618	1,032	1,691	3.52 × 10^7^
61	10	10	272,814	17,459	35,002	6,036	586	998	1,651	3.50 × 10^7^
61	20	20	154,459	15,906	25,114	4,575	545	891	1,408	2.24 × 10^7^
61	30	30	109,994	10,070	19,994	3,721	496	808	1,232	1.62 × 10^7^
61	50	50	70,029	10,349	13,916	2,675	455	725	1,073	1.01 × 10^7^
61	100	10	32,476	8,300	7,318	1,479	426	655	894	4.79 × 10^6^
61	100	100	32,392	7,822	7,231	1,463	423	652	893	4.72 × 10^6^
61	1000	1000	1,709	5,209	531	114	424	614	798	3.27 × 10^5^
71	2	2	1,431,412	15,641	131,742	22,422	360	617	870	8.13 × 10^7^
71	10	2	208,036	17,484	29,793	5,393	683	1,101	1,785	3.28 × 10^7^
71	10	10	219,099	17,432	31,400	5,567	629	1,041	1,705	3.27 × 10^7^
71	20	20	122,216	14,372	21,816	4,052	581	928	1,460	2.03 × 10^7^
71	30	30	86,599	10,416	17,138	3,249	524	835	1,272	1.43 × 10^7^
71	50	50	54,694	10,341	11,925	2,313	464	729	1,075	8.70 × 10^6^
71	100	10	23,980	7,817	6,183	1,253	424	650	892	4.02 × 10^6^
71	100	100	23,997	7,810	6,119	1,239	419	644	889	3.95 × 10^6^
71	1000	1000	1,199	5,202	443	87	444	660	885	2.93 × 10^5^
81	2	2	1,142,924	15,303	120,593	19,697	354	625	911	7.54 × 10^7^
81	10	2	158,781	17,713	24,939	4,721	788	1,202	1,898	3.00 × 10^7^
81	10	10	174,032	17,688	27,366	5,005	691	1,096	1,774	3.00 × 10^7^
81	20	20	92,627	15,784	17,715	3,396	654	1,011	1,573	1.79 × 10^7^
81	30	30	64,866	9,868	13,697	2,666	592	904	1,366	1.24 × 10^7^
81	50	50	39,613	10,328	9,358	1,874	513	778	1,130	7.28 × 10^6^
81	100	10	16,303	10,149	4,777	970	454	687	963	3.28 × 10^6^
81	100	100	16,493	10,155	4,817	981	438	671	943	3.23 × 10^6^
81	1000	1000	889	5,198	381	82	454	649	790	2.47 × 10^5^
91	2	2	932,237	15,694	108,954	17,394	344	622	936	6.79 × 10^7^
91	10	2	124,647	17,713	20,420	4,025	880	1,293	2,007	2.64 × 10^7^
91	10	10	142,306	17,687	23,525	4,428	727	1,126	1,804	2.65 × 10^7^
91	20	20	72,117	15,792	13,614	2,702	752	1,108	1,712	1.51 × 10^7^
91	30	30	48,540	15,792	9,949	2,009	700	1,023	1,529	1.02 × 10^7^
91	50	50	27,581	10,199	6,477	1,336	624	901	1,309	5.84 × 10^6^
91	100	10	10,503	10,105	3,081	658	564	816	1,155	2.52 × 10^6^
91	100	100	10,770	10,149	3,308	705	528	769	1,078	2.54 × 10^6^
91	1000	1000	598	3,008	342	76	438	621	754	2.12 × 10^5^

As part of our first approach, we also performed four independent *de novo* transcriptome assemblies with NGen: three with 20 million merged reads each and one with the remaining 12,114,709 merged reads (Table 2). We identified all full-length toxins from all four assemblies. Given that all three assembly methods tended to generate a large number of fragmented toxin sequences, apparently because of retained introns and possibly alternative splicing, we developed and implemented a simple hash-table approach to completing partial transcripts, which we will refer to as Extender (see Methods). We used Extender on partial toxin sequences identified for two of the four NGen assemblies. We also annotated the most abundant full-length nontoxin transcripts for the three assemblies based on 20 million reads. After combining all of the annotated toxin and nontoxin sequences from the ABySS, Velvet, and NGen assemblies and eliminating duplicates, we had 72 unique toxin sequences and 234 unique nontoxin sequences. The paucity of full-length annotated nontoxins reflects our focus on toxin sequences rather than their absence in the assemblies.

**Table 2:** NGen assembly summaries

			**No. contigs**	**Assembled**	**Unique full-**	**Unique Extender**
**Assembly**	**No. reads**	**No. contigs**	**> 2k**	**sequences**	**length toxins**	**toxins**
NGen 1	20,000,000	12,694	4,403	9,786,054	34	54
NGen 2	20,000,000	12,746	4,439	9,821,212	36	54
NGen 3	20,000,000	12,698	4,412	9,820,553	38	–
NGen 4	12,114,709	8,484	3,078	5,948,003	34	–
Total unique full-length toxins =						154

**Figure 2:**
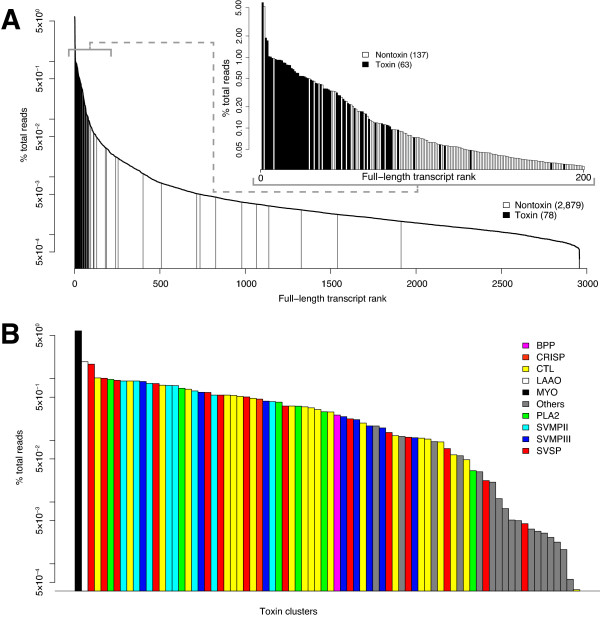
**Domination of the *C. adamanteus* venom-gland transcriptome by toxin transcripts.** The 123 unique toxin sequences were clustered into 78 groups with less than 1% nucleotide divergence for estimation of abundances. (**A**) The vast majority of the extremely highly expressed genes were toxins. The inset shows a magnification of the top 200 transcripts. (**B**) Expression levels of individual toxin clusters are shown with toxin classes coded by color. The toxin clusters are in the same order as in Table 3.

Our second approach to transcriptome assembly was designed to annotate as many full-length coding sequences (toxin and nontoxin) as possible and to build a reference database of sequences to facilitate the future analysis of other snake venom-gland transcriptomes. We found that NGen was much more successful at producing transcripts with full-length coding sequences but also that it was quite inefficient when the coverage distribution was extremely uneven (see Figure 2). Feldmeyer et al. [41] also found NGen to have the best assembly performance with Illumina data. We sought therefore first to eliminate the transcripts and corresponding reads for the extremely high-abundance sequences. To do so, we employed Extender as a *de novo* assembler by starting from 1,000 individual high-quality reads and attempting to complete their transcripts (see Methods). From 1,000 seeds, we identified 318 full-length coding sequences with 213 toxins and 105 nontoxins. After duplicates were eliminated, this procedure resulted in 58 unique toxin and 44 unique nontoxin full-length transcripts. These sequences were used to filter the corresponding reads from the full set of merged reads with NGen. We then performed a *de novo* transcriptome assembly on 10 million of the filtered reads with NGen, annotated full-length transcripts from contigs comprising ≥ 200 reads with significant blastx hits, and used the resulting unique sequences as a new filter. This process of assembly, annotation, and filtering was iterated two more times. The end result was 91 unique toxin and 2,851 unique nontoxin sequences.

**Figure 3:**
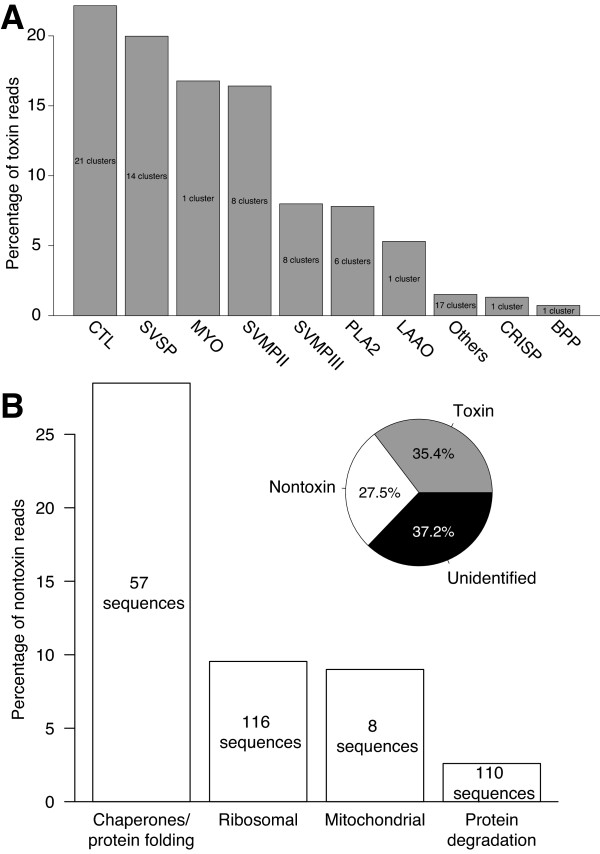
**Expression levels of major classes of toxins and nontoxins.** More than 60% of the total reads have been accounted for with full-length annotated transcripts. (**A**) The major toxin classes were the CTLs, SVSPs, MYO, and SVMPs (types II and III). (**B**) As expected for a protein-secreting tissue, the venom gland expresses an abundance of proteins involved in proteostasis.

The results from both assembly approaches were merged to yield the final data set. The first approach produced 72 unique toxin and 234 unique nontoxin sequences, and the second 91 toxin and 2,851 nontoxin sequences. The merged data set consisted of 123 unique toxin sequences and 2,879 nontoxins that together accounted for 62.9% of the sequencing reads (Figure 3).

### Toxin transcripts

We identified 123 individual, unique toxin transcripts with full-length coding sequences. To estimate the abundances of these transcripts in the *C. adamanteus* venom-gland transcriptome, we clustered them into 78 groups with less than 1% nt divergence (Table 3). Clusters could include alleles, recent duplicates, or even sequencing errors, which are characteristic of high-throughput sequencing [42]. For longer genes, clusters might also include different combinations of variable sites that are widely separated in the sequence. We chose 1% as a practical, but arbitrary, cut-off for defining clusters. Mapping reads back to more similar sequences to estimate abundances would be problematic because reads could not be uniquely assigned to a particular sequence. The true number of toxin genes for *C. adamanteus* probably lies somewhere between 78 and 123. This range is at the lower end of the number of unique toxins typically identified for viperids by means of proteomic techniques [10], which may indicate that the venom of *C. adamanteus* is less complex than that of other species. Alternatively, posttranscriptional processes such as alternative splicing or posttranslational modifications could significantly increase the diversity of toxins present in the venom. Our identified toxins accounted for 35.4% of the total reads (Figure 3), and the vast majority of the extremely high-abundance transcripts were those encoding toxin proteins (Figure 2A). We named toxins with a combination of a toxin-class abbreviation, a cluster number, and, if the cluster had more than a single member, a lower-case letter to indicate the member of the cluster (e.g., CTL-3b).

**Table 3:** Expression levels of full-length toxin clusters

**Rank**	**Cluster name**	**Cluster size**	**Length**	**% total reads**	**% toxin reads**	**GenBank TSA accessions**
1	MYO	1	994	5.93	16.780	JU173668
2	LAAO	1	3089	1.88	5.309	JU173667
3	SVSP-6	1	1720	1.71	4.849	JU173733
4	CTL-8	3	721	1.02	2.896	a:JU173656,
						b:JU173657,
						c:JU173658
5	SVSP-13	1	1661	1.01	2.864	JU173724
6	PLA2-1	2	904	9.68 × 10^−1^	2.739	a:JU173675,
						b:JU173676
7	SVSP-3	2	1830	9.38 × 10^−1^	2.653	a:JU173728,
						b:JU173729
8	SVMPII-5	8	2124	9.15 × 10^−1^	2.587	a:JU173694,
						b:JU173695,
						c:JU173696,
						d:JU173697,
						e:JU173698,
						f:JU173699,
						g:JU173700,
						h:JU173701
9	CTL-4	5	780	9.14 × 10^−1^	2.585	a:JU173646,
						b:JU173647,
						c:JU173648,
						d:JU173649,
						e:JU173650
10	SVMPII-1	5	2298	9.13 × 10^−1^	2.583	a:JU173682,
						b:JU173683,
						c:JU173684,
						d:JU173685,
						e:JU173686
11	SVMPIII-2	5	2246	8.97 × 10^−1^	2.538	a:JU173707,
						b:JU173708,
						c:JU173709,
						d:JU173710,
						e:JU173711
12	SVMPII-2	2	2138	8.38 × 10^−1^	2.369	a:JU173687,
						b:JU173688
13	SVSP-1	1	3120	8.30 × 10^−1^	2.348	JU173726
14	CTL-16	1	711	7.82 × 10^−1^	2.211	JU173631
15	SVMPII-7	1	2082	7.74 × 10^−1^	2.191	JU173703
16	SVMPII-3	4	1931	7.69 × 10^−1^	2.176	a:JU173689,
						b:JU173690,
						c:JU173691,
						d:JU173692
17	PLA2-4	1	890	6.98 × 10^−1^	1.974	JU173679
18	CTL-3	6	797	6.73 × 10^−1^	1.905	a:JU173640,
						b:JU173641,
						c:JU173642,
						d:JU173643,
						e:JU173644,
						f:JU173645
19	SVMPII-6	1	2183	6.31 × 10^−1^	1.784	JU173702
20	SVMPIII-3	3	2401	6.00 × 10^−1^	1.698	a:JU173712,
						b:JU173713,
						c:JU173714
21	SVSP-9	1	10270	5.96 × 10^−1^	1.685	JU173738
22	SVMPII-4	1	2016	5.41 × 10^−1^	1.530	JU173693
23	SVSP-8	1	3524	5.41 × 10^−1^	1.529	JU173737
24	CTL-7	1	763	5.40 × 10^−1^	1.528	JU173655
25	CTL-18	1	775	5.33 × 10^−1^	1.509	JU173633
26	CTL-1	1	763	5.15 × 10^−1^	1.458	JU173635
27	SVSP-12	1	957	5.04 × 10^−1^	1.424	JU173723
28	CTL-6	1	607	4.80 × 10^−1^	1.358	JU173654
29	CRISP	1	1579	4.66 × 10^−1^	1.317	JU173623
30	SVMPIII-7	1	2343	4.31 × 10^−1^	1.218	JU173719
31	SVMPII-8	1	1863	4.24 × 10^−1^	1.198	JU173704
32	PLA2-6	1	834	4.15 × 10^−1^	1.174	JU173681
33	SVSP-7	3	2108	3.59 × 10^−1^	1.016	a:JU173734,
						b:JU173735,
						c:JU173736
34	CTL-15	1	947	3.59 × 10^−1^	1.016	JU173630
35	PLA2-2	1	872	3.58 × 10^−1^	1.013	JU173677
36	CTL-10	1	587	3.49 × 10^−1^	0.987	JU173624
37	CTL-14	1	1246	3.36 × 10^−1^	0.951	JU173629
38	CTL-13	1	680	3.15 × 10^−1^	0.891	JU173628
39	PLA2-5	1	651	2.90 × 10^−1^	0.819	JU173680
40	CTL-9	2	755	2.88 × 10^−1^	0.814	a:JU173659,
						b:JU173660
41	BPP	1	1300	2.57 × 10^−1^	0.726	JU173621
42	SVMPIII-1	2	2665	2.41 × 10^−1^	0.683	a:JU173705,
						b:JU173706
43	SVSP-14	1	1732	2.23 × 10^−1^	0.631	JU173725
44	SVMPIII-4	2	2433	2.16 × 10^−1^	0.611	a:JU173715,
						b:JU173716
45	CTL-2	2	749	1.90 × 10^−1^	0.538	a:JU173638,
						b:JU173639
46	SVMPIII-8	1	2150	1.71 × 10^−1^	0.484	JU173720
47	VESP	1	1603	1.71 × 10^−1^	0.483	JU173741
48	SVMPIII-5	1	2339	1.59 × 10^−1^	0.449	JU173717
49	SVSP-4	2	2152	1.34 × 10^−1^	0.379	a:JU173730,
						b:JU173731
50	CTL-20	1	785	1.19 × 10^−1^	0.336	JU173636
51	NUC	1	2706	1.16 × 10^−1^	0.327	JU173671
52	SVSP-5	1	1890	1.12 × 10^−1^	0.317	JU173732
53	SVMPIII-6	1	2367	1.10 × 10^−1^	0.311	JU173718
54	CTL-21	1	824	1.09 × 10^−1^	0.307	JU173637
55	CTL-19	1	618	1.05 × 10^−1^	0.296	JU173634
56	NF	1	1395	9.53 × 10^−2^	0.269	JU173669
57	CTL-12	2	825	9.40 × 10^−2^	0.266	a:JU173626,
						b:JU173627
58	SVSP-2	1	1675	7.35 × 10^−2^	0.208	JU173727
59	CTL-5	3	637	5.83 × 10^−2^	0.165	a:JU173651,
						b:JU173652,
						c:JU173653
60	PDE	1	2743	5.62 × 10^−2^	0.159	JU173674
61	CTL-11	1	625	4.86 × 10^−2^	0.137	JU173625
62	PLA2-3	1	957	3.22 × 10^−2^	0.091	JU173678
63	CREGF	1	1945	3.09 × 10^−2^	0.087	JU173622
64	SVSP-10	1	1815	2.22 × 10^−2^	0.063	JU173721
65	HYAL-1	1	2545	2.10 × 10^−2^	0.059	JU173662
66	KUN	1	1698	1.14 × 10^−2^	0.032	JU173666
67	HYAL-2	1	1302	7.83 × 10^−3^	0.022	JU173663
68	KUN-1	1	2575	5.11 × 10^−3^	0.014	JU173664
69	VEGF-1	2	906	4.99 × 10^−3^	0.014	a:JU173739,
						b:JU173740
70	SVSP-11	1	1207	4.46 × 10^−3^	0.013	JU173722
71	GC	1	1730	3.65 × 10^−3^	0.010	JU173661
72	PDE-6	1	3691	3.36 × 10^−3^	0.010	JU173673
73	NGF	1	951	3.14 × 10^−3^	0.009	JU173670
74	KUN-2	1	1438	2.68 × 10^−3^	0.008	JU173665
75	PDE-4	1	2633	2.24 × 10^−3^	0.006	JU173672
76	VF	1	5087	1.70 × 10^−3^	0.005	JU173742
77	WAP	1	627	5.60 × 10^−4^	0.002	JU173743
78	CTL-17	1	774	3.80 × 10^−4^	0.001	JU173632

We used the number or percentage of reads mapping to a particular transcript as a measure of its abundance. Although average coverage might be a more appropriate proxy for the number of copies of a given transcript present, because it accounts for differences in transcript lengths, we prefer read counts as a measure of the expression expenditure on a given transcript because they better reflect the energetic cost associated with producing the encoded protein and are consistent with previous work using low-throughput sequencing (see, e.g., Pahari et al. [25]). In addition, this measurement should more closely match proteomic-based measurements of the contents of venom components (see, e.g., Gibbs et al. [43]) which come in the form of the percentages of total peptide bonds in the sample.

#### Snake venom metalloproteinases

We identified 39 unique sequences and 16 clusters of snake-venom metalloproteinases (SVMPs) that accounted for 24.4% of the reads mapping to toxin sequences and 8.6% of the total reads (Figure 3A and Table 3). In terms of total reads, the SVMPs were the most abundant class of toxins in the *C. adamanteus* venom-gland transcriptome. SVMPs are the primary sources of the local and systemic hemorrhage associated with envenomation by viperids and are divided into a number of subclasses based on their domain structure [44,45]. All SVMPs have a metalloproteinase domain characterized by a zinc-binding motif. All of the SVMPs identified for *C. adamanteus* belong to either the type II or the type III subclass. Type II SVMPs (SVMPIIs) have a disintegrin domain in addition to the metalloproteinase domain, which may be proteolytically cleaved posttranslationally to produce a free disintegrin. Type III SVMPs (SVMPIIIs) have a disintegrin-like and a cysteine-rich domain in addition to the metalloproteinase domain. We found 8 clusters of each of these two subclasses with 23 unique SVMPII sequences and 16 unique SVMPIII sequences. SVMPII and SVMPIII clusters comprise 16.4% and 8.0% of the reads mapping to toxins respectively (Figure 3). The sequences in both subclasses are diverse. The maximum pairwise nt divergence for the SVMPIIs was 10.0%, corresponding to a maximum amino-acid divergence of 18.1%. For the SVMPIIIs, the maximum pairwise nt divergence was 20.4% with a maximum amino-acid divergence of 42.3%. Although SVMPs were the dominant toxins as a class, the individual SVMP cluster with the highest abundance was SVMPII-5, which was only the eighth most abundant toxin cluster (Figure 2B and Table 3).

Mackessy [46] categorized rattlesnake venoms as type I or type II on the basis of their toxicities and metalloproteinase activities. These two measurements tend to be inversely related in rattlesnakes: species (or populations) with low LD_50_ values tend also to have low or undetectable hemorrhagic activities. SVMPs are the major hemorrhagic components of snake venoms, and high toxicity appears to be caused mostly by neurotoxic venom components. Low-toxicity venoms with high metalloproteinase activity are classified as type I, and high-toxicity venoms with low metalloproteinase activity are classified as type II. On the basis of the abundance of SVMPs in the venom-gland transcriptome, *C. adamanteus* clearly has type I venom, although the relatively low toxicity of its venom [46] is at least partially compensated for by its large size and venom yield.

#### C-type lectins

The most diverse and the second most abundant toxin class in the *C. adamanteus* venom-gland transcriptome was the C-type lectin (CTL) class. We identified 37 unique sequences and 21 clusters of CTLs that accounted for 22.2% of the reads mapping to toxins and 7.8% of the total reads (Figure 3A and Table 3). CTLs generally either inhibit or activate components of plasma or blood-cell types, thereby interfering with hemostasis [47]. Most known snake-venom CTLs function as heterodimers or even more complex arrangements [48], probably accounting in part for their diversity. The divergence among members of this class within the *C. adamanteus* genome was extreme, although all members preserved a CTL-like domain. Some pairs shared virtually no conserved amino-acid positions. Three of the CTL clusters provide evidence for the relevance of alternative splicing in the generation of toxin proteins. CTL-3f, CTL-4e, and CTL9b all have 48-nt insertions in the same region but are otherwise similar or identical to other members of their clusters.

#### Snake venom serine proteinases

The third most abundant toxin class for *C. adamanteus* was the snake-venom serine proteinases (SVSPs). We identified 18 unique sequences and 14 clusters in this toxin class, accounting for 20.0% of the toxin reads and 7.1% of the total reads (Figure 3A and Table 3). Three of the 10 most highly expressed individual toxins were SVSPs (Figure 2). SVSPs interfere with a wide array of reactions involving blood coagulation and hemostasis and belong to the trypsin family of serine proteases [49,50]. Mackessy [46] detected significant thrombin-like and kallikrein-like activity in the venom of *C. adamanteus*, which are attributable to the action of SVSPs. The diversity of SVSPs within the *C. adamanteus* genome is high; maximum pairwise nt divergence is 20.6% and amino-acid divergence is 47.4%.

The members of two SVSP clusters differ in a way that should be noted. The lengths of SVSPs are generally well conserved throughout the class. SVSP-7a has a 27-nt insertion relative to the two other members of its cluster but is otherwise identical to SVSP-7b. This difference could reflect the presence of alternative splicing for this gene. SVSP-3a is unique among the *C. adamanteus* SVSPs or those known from other snake species in apparently having a 65-amino-acid extension of its C-terminal region. The other member of its cluster, SVSP-3b, has a single deletion of a C nt in a poly-C tract that terminates its coding sequence consistently with other known SVSPs. The reads generating the SVSP-3a form vastly outnumber those for the SVSP-3b form; more than 95% of the reads support the extended version of the protein. The effect, if any, of this C-terminal extension remains to be determined.

#### Phospholipase A_2_’s

Previous work with *C. adamanteus* identified only a single phospholipase A_2_ (PLA2) sequence [11], but we identifed seven unique sequences in six clusters (Figure 2 and Table 3), accounting for 7.8% of the toxin reads and 2.8% of the total reads (Figure 3). PLA2s are among the most functionally diverse classes of snake-venom toxins and have pharmocological effects ranging from neurotoxicity (presynaptic or postsynaptic) to myotoxicity and cardiotoxicity. Anticoagulant and hemolytic effects due to PLA2s are also known [51,52]. Compared to other toxin classes of *C. adamanteus*, the diversity of PLA2s is low. Five of the six clusters are all within 5% nt divergence of one another. PLA2-3 is the lone, high-divergence outlier, differing by more than 31% at the nt level from the other clusters. PLA2-3 is also expressed at the lowest level of any of the PLA2s (Table 3).

#### Other high-abundance toxins

The SVMPs, CTLs, SVSPs, and PLA2s account for 74% of the reads mapping to toxin sequences (Figure 3), 73% of the toxin clusters, and 82% of the unique toxin sequences. The remaining toxins belong to 16 different classes. Many of these are low-abundance transcripts (Figure 2 and Table 3) and may not actually function as significant toxins, whereas several others have high to moderate abundances and represent significant components of the venom.

The most abundant toxin transcript and the most abundant transcript overall (Figure 2) was a small basic myotoxin related to crotamine [53,54]. The precursor protein is just 70 amino acids in length with a predicted 22-amino-acid signal peptide. This transcript was detected by Rokyta et al. [11], but the coding sequence was prematurely truncated in their sequence because of a single nt deletion. This toxin accounts for 16.8% of the toxin reads (Figure 3A) and 5.9% of the total reads. Crotamine, originally isolated from the venom of *C. durissus*, causes spastic paralysis in mice and is found in the venoms of many species of *Crotalus*[54]. Muscle spasms, twitching, and paralysis of the legs have been reported for human envenomations by *C. adamanteus*[20]. Interestingly, Straight et al. [55] noted that individuals of *C. adamanteus* from populations in southern and central Florida lack this toxin in their venoms. Given that this myotoxin is the most abundant transcript in the venom of our specimen, its absence in southern populations points to a dramatic difference in venoms within this species and the potential for significantly different pathological effects associated with bites from different *C. adamanteus* populations.

A single L-amino-acid oxidase (LAAO) transcript was the second most abundant toxin transcript (Figure 2B), consistent with the previously detected LAAO activity in the *C. adamanteus* venom [46]. This single transcript accounted for 5.3% of the reads mapping to toxins and 1.9% of the total reads. LAAOs are flavoproteins, giving the venom its yellow color; can be edema- or apoptosis-inducing; and can induce or inhibit platelet aggregation [56]. These effects are probably mediated by H_2_O_2_released during the oxidation reaction catalyzed by the enzyme. The 29th most abundant toxin transcript was a cysteine-rich secretory protein (CRISP) (Figure 2B and Table 3), accounting for 1.3% of the toxin reads (Figure 3A). Although CRISPs are widely found in snake venoms, their precise effects are not well established [57], but they appear to interfere with smooth-muscle contraction [58,59]. A single transcript for a bradykinin-potentiating and C-type natriuretic peptide transcript (BPP) was found to account for 0.7% of the toxin reads (Figure 3A). The encoded protein is similar to a protein identified in *Sistrurus catenatus* (GenBank accession: DQ464265) that was hypothesized to reduce blood pressure in envenomated prey [25]. A loss of blood pressure has been reported in human envenomations by *C. adamanteus*[20].

#### Other low-abundance toxins

The remaining 17 clusters are classified as “others” in Figure 3A. Because each has a relatively low expression level (Table 3), many of these should be considered putative toxins until their presence in the *C. adamanteus* venom is confirmed proteomically and pharmacological effects are associated with them.

Rokyta et al. [11] detected the presence of a transcript encoding a protein homologous to ohanin from *Ophiophagus hannah*[60,61] and to a homologous protein from *Lachesis muta*[62]; we found a transcript identical to that of Rokyta et al. [11]. Pung et al. [60,61] found the *O. hannah* version of this protein to increase pain sensitivity (hyperalgesia) and to induce temporary hypolocomotion in mice and proposed naming the class vespryns (VESP). Exceptionally intense pain has been reported after envenomation of humans by *C. adamanteus*[20], although whether such pain is due to a specific toxin is not clear.

We detected three different nucleotidases (NUCs) and five different phosphodiesterases (PDEs) in the venom-gland transcriptome of *C. adamanteus*. Only one of the NUCs and three of the PDEs had signal peptides, and we therefore only considered these as potential toxins: NUC, PDE, PDE-4, and PDE-6 (Table 3). The roles of these enzymes in venoms are uncertain, but their primary function may be to liberate toxic nucleosides [63-65]. Significant PDE activity has been detected previously in the venom of *C. adamanteus*[46].

The *C. adamanteus* venom-gland transcriptome contained three Kunitz-type protease inhibitors (KUNs). Two of these shared more than 75% animo-acid identity with a KUN from *Austrelaps labialis* (GenBank accession: B2BS84), an Australian elapid. All three KUNs have domains that place them in the superfamily of bovine pancreatic trypsin-like inhibitors, and snake toxins from this family are known to inhibit plasma serine proteinases. Although KUNs are commonly observed in snake venoms, their role in envenomation (if any) is not well defined [66]. The three KUNs detected for *C. adamanteus* are all at relatively low abundances, suggesting that they are not major components of the venom.

We identified two transcripts, HYAL-1 and HYAL-2, encoding hyaluronidase-like proteins. Hyaluronidases are generally regarded as venom components that promote the dissemination of other venom components by degrading the extracellular matrix at the site of injection [67], although they may have more direct toxic effects [68]. The coding sequences of our two transcripts differ only in the presence of a 765-nt deletion in HYAL-2 relative to HYAL-1. Truncated hyaluronidases such as HYAL-2 have been detected in the venoms of other viperid species [67] and may represent an example of alternative splicing. We also identified a transcript encoding a glutaminyl-peptide cyclotransferase (glutaminyl cyclase; GC). Many snake venom components have N termini blocked by pyroglutamate, and GCs catalyze the formation of this block. This component is related more to maturation and protection of other toxins and probably contributes only indirectly to toxicity [69].

We identified six growth-factor-related sequences in the venom-gland transcriptome of *C. adamanteus*: a nerve growth factor (NGF), a neurotrophic factor (NF), two vascular endothelial growth factors (VEGF) in a single cluster, and a cysteine-rich with EGF-like domain protein (CREGF). The NGF transcript encodes a 241 amino-acid precursor protein and shares 99% amino-acid identity with a NGF from *C. durissus* (GenBank accession: AAG30924). The NF transcript encodes a 180-amino-acid precursor that shares homology with mesencephalic astrocyte-derived neurotrophic factors. We found no close venom-related sequences for this NF in the available databases. The VEGF sequences appear to be alternatively spliced versions of one another. VEGF-1a encodes a 192-amino-acid precursor, and VEGF-1b encodes a 148-amino-acid precursor. Aside from the 132-nt deletion in VEGF-1b relative to VEGF-1a, their coding sequences are identical. Both forms have database matches of the same length with 99% amino-acid identity from *Trimeresurus flavoviridis* (GenBank accessions: AB154418 and AB154419). Finally, we detected the same cysteine-rich with EGF-like domain protein as described by Rokyta et al. [11].

The final two putative toxin transcripts are of questionable significance because of their low expression levels. A single sequence with 77% amino-acid identity to a waprin (WAP) sequence from *Philodryas olfersii* (GenBank accession: EU029742), a rear-fanged colubrid, was detected. Related sequences have been detected in a variety of other rear-fanged snake species, but such proteins are only known to exhibit antimicrobial activity [70]. We detected a venom factor (VF) transcript that shares 87% animo-acid identity with a VF from *Austrelaps superbus* (GenBank accession: AY903291) [71]. The *C. adamanteus* VF transcript encodes a 1,652-amino-acid precursor with a 22-amino-acid signal peptide. The best-studied member of this toxin family is cobra venom factor, which is known to activate the complement system [72]. The extremely low expression levels of these transcripts may indicate that they represent the orthologous genes to the ancestors of the known toxic forms and may therefore have no toxic functions.

#### Comparison to previous work

Rokyta et al. [11] previously described toxin transcripts in the venom-gland transcriptome of *C. adamanteus* on the basis of 454 pyrosequencing. Their work used RNA from the venom gland of the same individual used in the present work. They found 40 unique toxin transcripts, 10 of which contained only partial coding sequences. Table 4 lists the closest matches from our current sequences to those of Rokyta et al. [11]. The vast majority of the 454-based sequences had either identical matches in our current set of toxins or matches with less than 1% nt divergence (Table 4). Only a single 454 toxin, SVSP-9, did not have a close match. This sequence contains only a partial coding sequence and therefore may not represent a true, functional toxin.

**Table 4:** **Correspondence with the results of Rokyta et al.**[11]**]**

**454 name**	**Accession**	**Closest match**	**% nt divergence**	**Notes**
CREGF	HQ414087	CREGF	0.1	
CRISP	HQ414088	CRISP	0.0	Identical
CTL-1	HQ414089	CTL-4a	0.0	Identical
CTL-2	HQ414090	CTL-8a	0.0	Identical
CTL-3	HQ414091	CTL-1	0.0	Identical
CTL-4	HQ414092	CTL-9a	0.8	
CTL-5	HQ414093	CTL-3e	0.9	
CTL-6	HQ414094	CTL-12b	0.0	Identical
CTL-7	HQ414095	CTL-10	0.0	Identical
CTL-8	HQ414096	CTL-2a	0.0	Identical
CTL-9	HQ414097	CTL-5a	0.0	Identical
HYAL	HQ414098	HYAL-1	0.0	454 version incomplete
LAAO	HQ414099	LAAO	0.0	Identical
MYO	HQ414100	MYO	0.0	454 version has 1-nt deletion that truncates the coding sequence prematurely
NUC	HQ414101	NUC	0.0	454 version incomplete
PDE-1	HQ414102	PDE	0.0	454 version has 123-nt insertion
PDE-2	HQ414103	PDE-2 (nontoxin)	0.0	454 version incomplete; no signal peptide; no longer considered toxin
PLA2	HQ414104	PLA2-1b	0.0	Identical
PLB	HQ414105	PLB (nontoxin)	0.2	No longer considered toxin
SVMP-1	HQ414106	SVMPII-3b	0.0	Identical
SVMP-2	HQ414107	SVMPII-3b/c	0.5	
SVMP-3	HQ414108	SVMPII-5a	0.3	
SVMP-4	HQ414109	SVMPIII-2d	1.2	
SVMP-5	HQ414110	SVMPIII-4b	1.0	
SVMP-6	HQ414111	SVMPIII-2d	0.2	
SVMP-7	HQ414112	SVMPIII-4a	0.0	Identical
SVMP-8	HQ414113	SVMPIII-5	0.5	454 version incomplete
SVMP-9	HQ414114	SVMPIII-1a/b	0.0	454 version incomplete
SVMP-10	HQ414115	SVMPIII-6	0.0	454 version incomplete
SVMP-11	HQ414116	SVMPIII-3a	0.0	454 version incomplete
SVSP-1	HQ414117	SVSP-3a	0.0	454 version has 1-nt deletion that truncates the coding sequence prematurely
SVSP-2	HQ414118	SVSP-1	0.0	Identical
SVSP-3	HQ414119	SVSP-7a	0.0	Identical
SVSP-4	HQ414120	SVSP-5	0.1	
SVSP-5	HQ414121	SVSP-9	0.5	
SVSP-6	HQ414122	SVSP-6	0.0	Identical
SVSP-7	HQ414123	SVSP-4b	0.0	454 version incomplete
SVSP-8	HQ414124	SVSP-2	0.0	454 version incomplete
SVSP-9	HQ414125	None	>10	454 version incomplete
VESP	HQ414126	VESP	0.0	Identical

### Nontoxin transcripts

**Figure 4:**
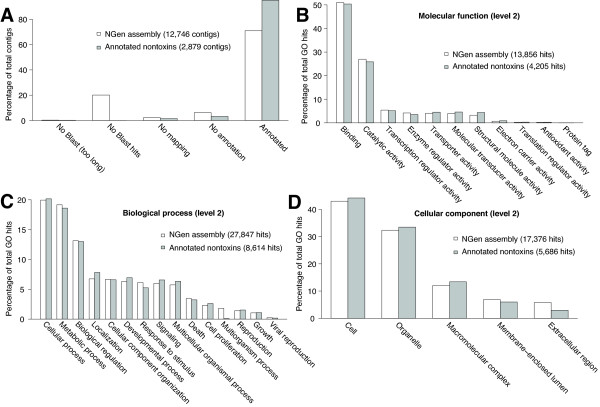
**Comparison of gene ontology (GO) results for our annotated full-length nontoxin sequences with those of the contigs from a*de novo* assembly with NGen.** Only level 2 GO terms are shown. The distributions of GO terms are similar across data sets, suggesting that the annotated transcripts provided a comprehensive characterization of the genes expressed in the venom gland. (**A**) The distributions of sequences reaching various stages of identification and annotation are shown. The level 2 GO terms are shown for molecular function (**B**), biological process (**C**), and cellular component (**D**).

We characterized the nontoxin genes expressed in the *C. adamanteus* venom gland by two means. First, we took all of the contigs from one of our four *de novo* NGen assemblies based on 20 million merged reads and conducted a full Blast2Go [73] analysis on the contigs comprising ≥ 100 reads. Of the 12,746 contigs (assembly 2 in Table 2), we were able to provide gene ontology (GO) annotations for 9,040 of them (Figure 4A). The major functional classes (level 2) represented in these results were binding and catalysis, followed by transcription regulation (Figure 4B). The major biological process GO terms (level 2) were cellular processes and metabolic processes (Figure 4C). Interestingly, viral reproductive function was detected and probably represents the activity of transposable elements or retroviruses like those previously noted in snake venom-gland transcriptomes [34]. The major cellular component GO terms (level 2) were cell and organelle (Figure 4D). For these results, we made no attempt to exclude toxin sequences, because they are necessarily a small minority of the total sequences, and did not require that contigs contain full-length coding sequences.

**Figure 5:**
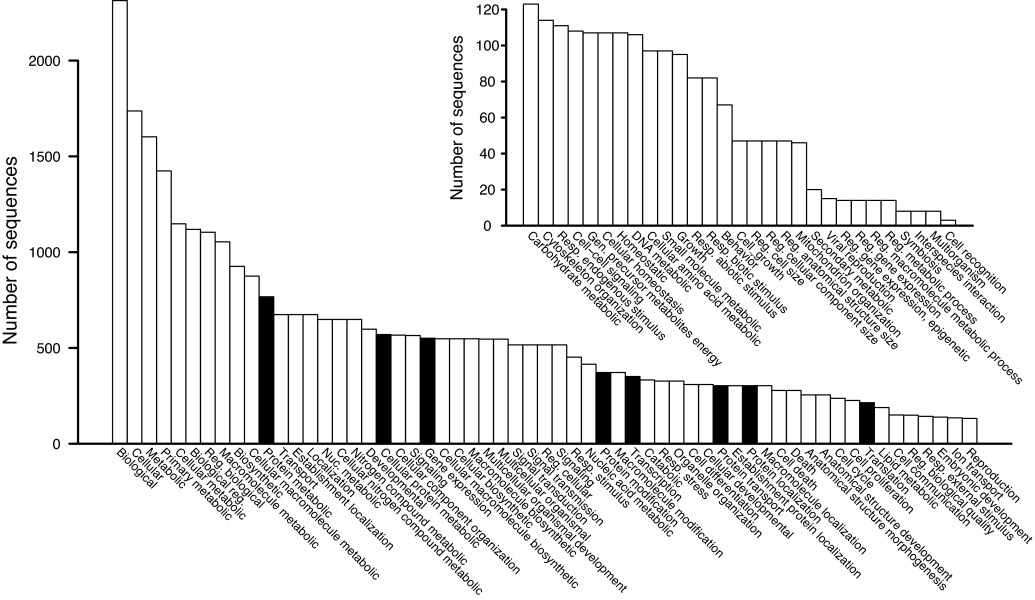
**The biological-process GO terms identified for the 2,879 annotated full-length nontoxin sequences.** Terms specific for the production, processing, and export of proteins are highlighted in black. The inset shows the low-abundance portion of the full distribution.

**Figure 6:**
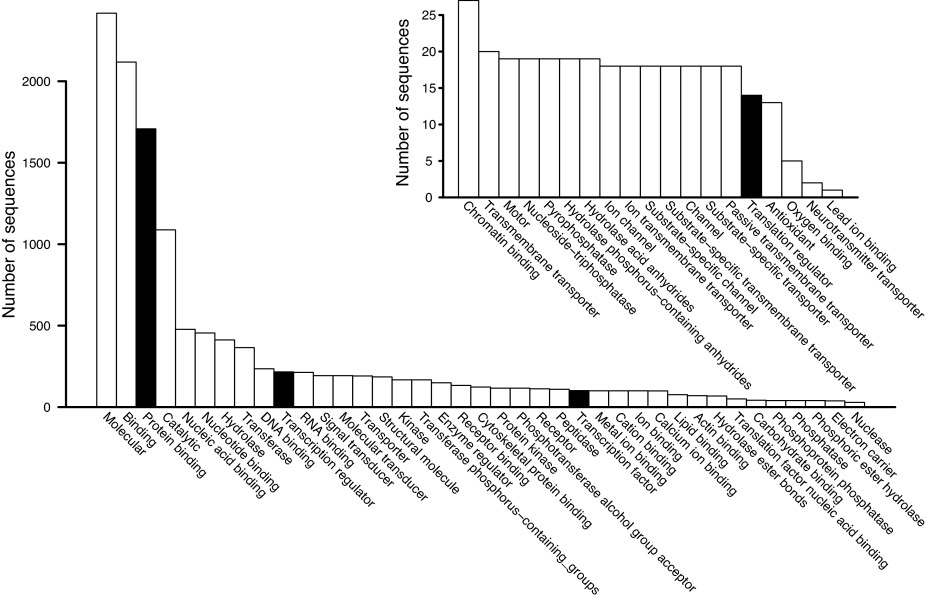
**The molecular-function GO terms identified for the 2,879 annotated full-length nontoxin sequences.** Terms specific for the production, processing, and export of proteins are highlighted in black. The inset shows the low-abundance portion of the full distribution.

**Figure 7:**
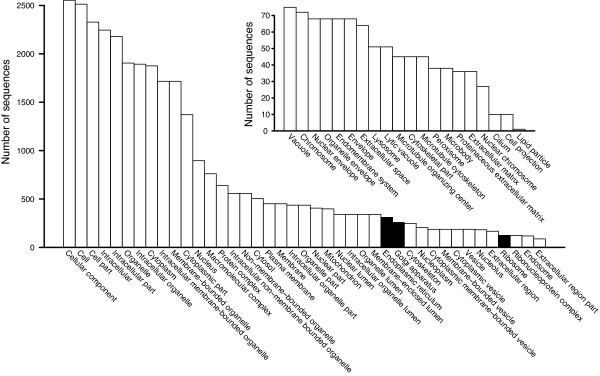
**The cellular-components GO terms identified for the 2,879 annotated full-length nontoxin sequences.** Terms specific for the production, processing, and export of proteins are highlighted in black. The inset shows the low-abundance portion of the full distribution.

For our second approach, we used only the 2,879 transcripts with full-length coding sequences for nontoxin proteins. We analyzed these sequences with Blast2GO. The distributions of level 2 GO terms for these data were almost identical to those of the full NGen assembly described above (Figure 4), suggesting that our 2,879 annotated nontoxin sequences provide a representative sample of the full venom-gland transcriptome. The full distributions of GO terms for these sequences across all levels are shown in Figures 5, 6, and 7. As expected for a secretory tissue, processes related to protein production and secretion were well represented (e.g., protein transport and protein modification; Figure 5), as were protein-binding functions (Figure 6) and proteins localized to the endoplasmic reticulum (ER) and the Golgi apparatus (Figure 7).

Four of the top 20 most highly expressed nontoxin genes (Table 5), including the most highly expressed, were protein disulfide isomerases (PDIs). In particular, they were members of the PDI family that is retained in the ER and are characterized by having two or more PDI domains, which are similar to thioredoxin. PDIs catalyze the formation or breaking of disulfide bonds and are therefore involved in protein folding. Molecular chaperones were well represented in the top 20 nontoxins by four genes: endoplasmin (a member of the HSP90 family), calreticulin, 78-kDA glucose-regulated protein (GRP78), and heat shock protein 5. The latter gene appears to be a splice variant of GRP78, differing within the coding region by two point mutations and two short deletions. All of these chaperones are ER specific. Six of the top 20 nontoxins were mitochondrial genes involved in oxidative cellular respiration, consistent with the high energetic demands of venom production [74]: cytochrome C oxidase subunits I and III, cytochrome B, and NADH dehydrogenase subunits 1, 4, and 5. The cells of venom glands are particularly rich in mitochondria [75]. Four genes were involved in various aspects of translation: two translation elongation factors, 18S rRNA, and vigilin. Vigilins are hypothesized to be involved in regulating mRNA stability and translation and might be involved in RNA-mediated gene silencing [76,77]. The final top 20 nontoxin gene was actin, a component of the cytoskeleton.

**Table 5:** The 20 most highly expressed nontoxin transcripts

**Name**	**Length**	**% reads**	**Function**	**Accession**											
Protein disulfide isomerase	2970	5.223	Rearrange disulfide bonds (ER)	JU175360											
Cytochrome C oxidase subunit I	2789	0.966	Electron transport chain	JU175042											
Cytochrome B	1275	0.499	Electron transport chain	JU175040											
Translation elongation factor 1 *α* 1	1985	0.459	Translation	JU174424											
18S rRNA	2509	0.421	Ribosomal component	JU173759											
Calreticulin	1661	0.406	Protein chaperone (ER)	JU174061											
Endoplasmin (HSP90 family)	3012	0.333	Protein chaperone (ER)	JU174456											
78 kDa glucose-regulated protein	2676	0.332	Protein chaperone (ER)	JU174713											
Heat shock protein 5 (GRP78 splice variant?)	2063	0.327	Protein chaperone (ER)	JU174801											
NADH dehydrogenase subunit 5	4448	0.272	Electron transport chain	JU175113											
Cytochrome C oxidase subunit III	2103	0.239	Electron transport chain	JU175043											
Protein disulfide isomerase A6	4933	0.212	Rearrange disulfide bonds (ER)	JU175358											
Nucleobindin 2	2937	0.203	Calcium binding	JU175278											
Protein disulfide isomerase A3	1904	0.186	Rearrange disulfide bonds (ER)	JU175355											
NADH dehydrogenase subunit 1	1878	0.173	Electron transport chain	JU175111											
NADH dehydrogenase subunit 4	1751	0.172	Electron transport chain	JU175112											
Protein disulfide isomerase A4	2540	0.159	Rearrange disulfide bonds (ER)	JU175356											
Translation elongation factor 2	3057	0.147	Translation	JU174429											
Vigilin 2	6107	0.129	mRNA stability and translation	JU176512											
Actin, cytoplasmic 2	1971	0.124	Cytoskeleton	JU173777											

The abundances of several major classes of nontoxins are provided in Figure 3B. We identified 57 sequences with functions related to protein folding [19,78-80], including various classes of heat-shock proteins, protein-disulfide isomerases, peptidyl-prolyl cis-trans isomerases, dnaJ-complex components, and T-complex components. These sequences together accounted for 28.4% of the total reads mapping to nontoxins. Ribosomal-protein transcripts (cytoplasmic and mitochondrial) accounted for 9.5% of the nontoxin reads, and mitochondrial genes accounted for another 9.0%. Finally, we identified 110 sequences transcripts encoding proteins involved in protein degradation [81,82], including proteins involved in the ubiquitin-proteasome system and the ER-associated protein-degradation system [83], which accounted for 2.6% of the nontoxin reads. Protein-quality control should be essential in a high-throughput protein-producing tissue such as a snake venom gland.

Our collection of nontoxins included several notable potential inhibitors of the toxins or other proteases (Table 6). Such inhibitors may play a role in preventing autolysis [84] or may serve to protect venom components once inside a victim [85]. We detected three cystatin-like transcripts in the venom gland. Cystatins are cysteine-protease inhibitors and have been detected in numerous elapid venom glands and venoms [85]. We detected three unique metalloproteinase inhibitors and two serine proteinase inhibitors (serpins). Finally, we found four unique PLA2 inhibitors.

**Table 6:** Toxin and protease inhibitors detected in the venom-gland transcripts

**Name**	**Length**	**% reads**	**Function**	**Accession**										
Cystatin 1	790	9.80 × 10^−4^	Cysteine-protease inhibitor	JU174278										
Cystatin B	460	1.23 × 10^−3^	Cysteine-protease inhibitor	JU174279										
Cystatin 2	709	1.31 × 10^−3^	Cysteine-protease inhibitor	JU174280										
Metalloproteinase inhibitor 1	820	1.04 × 10^−3^	Metalloproteinase inhibitor	JU175124										
Metalloproteinase inhibitor 2	2560	2.57 × 10^−3^	Metalloproteinase inhibitor	JU175125										
Metalloproteinase inhibitor 3	2202	1.01 × 10^−3^	Metalloproteinase inhibitor	JU175126										
PLA2 inhibitor beta	1210	1.54 × 10^−3^	PLA2 inhibitor	JU175425										
PLA2 inhibitor gamma B 1	1492	3.68 × 10^−3^	PLA2 inhibitor	JU175444										
PLA2 inhibitor gamma B 2	1694	7.70 × 10^−4^	PLA2 inhibitor	JU175442										
PLA2 inhibitor B	2339	1.07 × 10^−3^	PLA2 inhibitor	JU175443										
Serpin B6	1708	9.68 × 10^−3^	Serine-proteinase inhibitor	JU175869										
Serpin H1	2004	9.40 × 10^−4^	Serine-proteinase inhibitor	JU175870										

### Sequence accession numbers

The original, unmerged sequencing reads were submitted to the National Center for Biotechnology Information (NCBI) Sequence Read Archive under accession number SRA050594. The annotated toxin and nontoxin sequences were submitted to the GenBank Transcriptome Shotgun Assembly (TSA) database under accession numbers JU173621–JU173743 (toxins) and JU173744–JU176622 (nontoxins).

## Conclusions

We have described the most comprehensive venom-gland transcriptomic characterization of a snake species to date and provided full-length coding sequences for 123 unique toxin proteins and 2,879 unique nontoxin proteins. We have demonstrated the use of Illumina sequencing technology for the sequencing and *de novo* assembly of a tissue-specific transcriptome for a nonmodel species, *C. adamanteus*, for which genome-scale resources were previously unavailable. Because the nontoxin sequences in particular should be conserved across snake species, our results should greatly facilitate similar work with other venomous species, serving as an assembly template and reducing the number of reads for which *de novo* assembly will be necessary.

The expressed toxin genes in the venom gland of *C. adamanteus* provide a detailed portrait of a type I rattlesnake venom [46]. The most abundant transcript expressed in the *C. adamanteus* venom gland encoded a myotoxin homologous to crotamine. Crotamine is known to induce spastic paralysis [54], a symptom that has been observed in human envenomations by *C. adamanteus*[20]. Like those of most viperids, the bites of *C. adamanteus* result in significant tissue damage and necrosis, and we found that SVMPs, the major class of hemorrhagic toxins, dominated venom-gland gene expression. The second most abundant toxin transcript overall was an LAAO, which are also noted for causing local tissue damage [46]. Coagulopathy is a common occurrence with pit-viper bites [5]. The CTLs and SVSPs were also both diverse and abundant in the venom-gland transcriptome of *C. adamanteus*, and both classes primarily attack the hemostatic system. In terms of gene sequences of venom components, the venom of *C. adamanteus* is now the best-characterized snake venom, although a thorough proteomic analysis of the venom is still needed. The sequences we have generated will greatly facilitate such a proteomic characterization by serving as a database against which to query mass-spectrum results.

The expression patterns of the nontoxin genes in the venom gland of *C. adamanteus* reflect the protein-secretory function of the tissue and the high energetic demands of rapid venom production [75]. The most highly expressed nontoxin genes were those involved in the production and processing of proteins and energy production to support these activities. Molecular chaperones and PDIs were particularly abundant. Though the expression patterns for nontoxins were not surprising, future comparisons with other snake species, especially those from other snake families, may be able to elucidate the origin and early stages of the evolution of the venom gland.

## Methods

### Venom-gland transcriptome sequencing

We sequenced the venom-gland transcriptome of a single animal from Florida (Wakulla County): an adult female weighing 393 g with a snout-to-vent length of 792 mm and a total length of 844 mm. To stimulate transcription in the venom glands, we anesthetized the snake by propofol injection (10 mg/kg) and extracted venom by electrostimulation under anesthesia [86]. After venom extraction, the animal was allowed to recover for four days while transcription levels reached their maxima [87]. The snake was euthanized by injection of sodium pentobarbitol (100 mg/kg), and its venom glands were subsequently removed. The above techniques were approved by the Florida State University Institutional Animal Care and Use Committee (IACUC) under protocol #0924.

Sequencing and nonnormalized cDNA library preparation were performed by the HudsonAlpha Institute for Biotechnology Genomic Services Laboratory (http://www.hudsonalpha.org/gsl/). Transcriptome sequencing was performed essentially as described by Mortazavi et al. [88] in a modification of the standard Illumina methods described in detail in Bentley et al. [89]. Total RNA was reduced to poly-A+ RNA with oligo-dT beads. Two rounds of poly-A+ selection were performed. The purified mRNA was then subjected to a mild heat fragmentation followed by random priming for first-strand synthesis. Standard second-strand synthesis was followed by standard library preparation with the double-stranded cDNA as input material. This approach is similar to that of Illumina’s TruSeq RNA-seq library preparation kit. Sequencing was performed in one lane on the Illumina HiSeq 2000 with 100-base-pair paired-end reads.

### Transcriptome assembly and analysis

The average insert length of our cDNA library was ∼170 nt, excluding the Illumina adaptors. With 100-base-pair paired-end sequencing, the majority of paired-end reads overlapped at their 3’ ends. Because read quality declines toward the 3’ ends of reads, we developed a method similar to that of Rodrigue et al. [36] for merging the overlapping pairs into single, long, high-quality reads. The members of each pair of reads were slid along each other, and, for each overlap of length *n*, we calculated the probability of getting the observed number of matches *k* by chance using a binomial probability given by

(1) $$P(k|n)=\left(\begin{array}{c} n\\ k \end{array}\;\right){\left(\frac{1}{4}\right)}^{k}{\left(\frac{3}{4}\right)}^{n-k}$$

assuming any of the four nucleotides is equally likely to be at any position. To be conservative, we only merged reads if the minimum probability was less than 10^−10^and the second smallest probability was at least 1000 times larger (Figure 1A). The latter condition was meant to help avoid merging reads that span highly repetitive regions. For cases in which the insert size was less than the read length, sequence data outside the overlap were assumed to represent adaptors and were deleted. We updated quality scores for the overlapping positions following the approach of Rodrigue et al. [36]. For merged reads, quality scores for nonoverlapping bases were left unchanged (Figure 1B). The unmerged reads were typically those pairs from the longer end of the insert-size distribution.

Because of the inherent difficulty in *de novo* transcriptome assembly, we used a diverse array of assembly approaches and combined the results for a final data set. We performed assemblies using ABySS version 1.2.6 [37,38] under a wide array of parameter values using both the merged and unmerged reads. In particular, we used *k*-mer values of 51, 61, 71, 81, and 91 and varied the coverage (*c*) and erode (*e*) parameters from 2 to 1,000. We set *E* = 0, *m* = 20, and *s* = 200 for all assemblies. Trans-ABySS [90] provided little or no improvement of our assemblies, primarily because assembly quality appeared to be more dependent on the coverage and erode parameters than on the *k*-mer length. We also conducted assemblies using both the merged and unmerged reads with Velvet version 1.1.02 [39] and *k*-mer values of 71, 81, and 91. We selected the best of these assemblies on the basis of the *N*50 values for further assembly into transcripts with Oases version 0.1.20 (http://www.ebi.ac.uk/∼zerbino/oases/) [40]. For Oases, we set the minimum transcript length to 300 nt and the coverage cutoff to 10. We also followed the approach of Rokyta et al. [11] and used the NGen2.2 assembler from DNAStar (http://www.dnastar.com/). Because this assembler is limited to 20–30 million reads, we used only the merged reads. We performed four independent assemblies: three with 20 million merged reads each and one with the remaining 12,114,709 merged reads. Each assembly was performed with the default settings for high-stringency, *de novo* transcriptome assembly for long Illumina reads, including default quality trimming. The high-stringency setting corresponded to setting the minimum match percentage to 90%. We retained contigs comprising at least 100 reads.

In addition to the all-at-once assembly approaches above, we developed an iterative approach that was both more effective at generating full-length transcripts and more computationally efficient. The first step consisted of applying our Extender program (see below) as a *de novo* assembler starting from 1,000 reads. Full-length transcripts were identified with blastx searches (see below), then used as templates in a reference-based assembly in NGen3.1 with a 98% minimum match percentage to filter reads corresponding to identified transcripts. Ten million of the unassembled sequences were then used in a *de novo* transcriptome assembly in NGen3.1 with the same settings as described above for *de novo* assembly except that the minimum match percentage was increased to 93% and contigs comprising less than 200 sequences were discarded. The resulting sequences were identified, where possible, by means of blastx searches, and the identified full-length transcripts were used in another templated assembly to generate a further-reduced set of reads. This iterative process was repeated two additional times.

To provide transcriptional profiles of the venom gland, we performed GO annotation with Blast2GO [73]. We ran full analyses on one of NGen assemblies of 20 million merged reads, including blastx searches, GO mapping, and annotation. We used the default Blast2GO parameters throughout. We converted the GO annotation to generic GO-slim terms. We ran the same analysis on the combined set of annotated nontoxin sequences.

For gene identification and annotation, we conducted blastx searches using mpiblast version 1.6.0 (http://www.mpiblast.org/) of the consensus sequences of contigs of our assemblies against the NCBI nonredundant protein database (nr; downloaded March 2011 and updated through November 2011). We used an E-value cut-off of 10^−4^, and only the top 10 matches were considered. For toxin identification, hit descriptions were searched for a set of keywords based on known snake-venom toxins and protein classes. Any sequence matching these keywords was checked for a full-length coding sequence. We generally only retained transcripts with full-length coding sequences (but see below). For the iterative assembly approach, the remaining, presumably nontoxin-encoding, contigs were screened for those whose match lengths were at least 90% of the length of at least one of their database matches. This step was intended to minimize the number of fragmented or partial sequences that were considered for annotation. In addition, we sorted the contigs of the three 20-million-sequence NGen assemblies from the all-at-once approach on the basis of the number of reads and attempted to annotate the top 500 contigs from one assembly and the top 100 from the other two.

We estimated transcript abundances using high-stringency reference-based assemblies in NGen3.1 with a minimum match percentage of 95. Ten million of the merged reads were mapped onto the full-length, annotated transcripts, and the percentage of reads mapping to each transcript was used as a proxy for abundance.

### The extender

The purpose of Extender is to estimate quickly one or more full-length transcript sequences from a large number of high-quality sequence reads. The procedure begins with one or more seed sequences provided by the user. The seeds can be known sequences (e.g., partial transcripts from a previous assembly) or simply sequences of one or more of the reads. The Extender procedure begins by hashing the *k*-mers observed at the two ends of the seeds. If *k* is set to 50, for example, then the 50-base sequence present at the 5’ end of each seed is used as a key in a hash table, and the hash value is a pointer to the seed in the list of seeds. A second hash table is likewise used for *k*-mers from the 3’ ends of the seeds. Note that this method requires that all initial *k*-mers be unique (that no two sequence ends be identical). Once the seeds are hashed, the seeds are extended with the set of reads provided by the user as follows. The two *k*-mers from the ends of each read are looked up in each hash table. If the key is present in the hash table, the seed is extended by concatenation of the nonoverlapping bases from the read onto the appropriate end of the seed. If the key is absent, the reverse complement of the read is used to extend the seed if the end *k*-mers are found. After each extension, the *k*-mer key facilitating the extension is removed from the hash table and the new *k*-mer key is added (the reference to the seed remains the same). The procedure is repeated until the reads have been cycled through *N* times, where *N* is chosen by the user. Cycling is beneficial because the Extender does not reset to the beginning of the read list when an extension is made.

Extension of a seed typically terminates when the end of the full-length transcript is reached or when a sequencing error is encountered in the end of an incorporated read. The presence of low-frequency biological artifacts (e.g., unspliced introns) may also result in termination of the extension. In order to improve the accuracy of the consensus sequence prediction, Extender can create replicate seeds for a particular seed by sequentially trimming one base at a time from both ends. Using replicate seeds allows several independent sequences that represent the same target consensus sequence to be generated simultaneously, and these replicates are entirely independent because they begin with different keys. The user can obtain the final estimate of the sequence corresponding to each original seed by taking the consensus across replicates or by simply choosing the replicate producing the longest sequence. We took the former approach for all of our assembly efforts. Overall, Extender is highly inefficient with its use of data and requires many long, high-quality reads, but it is extremely computationally efficient, having short run times and low memory requirements.

We used Extender in two different ways: to complete partial toxin transcripts and as a *de novo* assembler. For the former, we used partial toxin transcripts from NGen assemblies that were found to have fragments of coding sequence homologous with known toxins. The partial transcripts were trimmed to just the partial coding sequence and used as seeds. To use Extender as a *de novo* assembler, we seeded it with 1,000 random reads. For both applications, we used a *k*-mer size of 100, 20 replicates, 10 cycles through the complete set of merged reads excluding all reads with any bases with quality scores less than 30.

## Abbreviations

BPP: Bradykinin potentiating and C-type natriuretic peptides; CTL: C-type lectin; CREGF: Cysteine-rich with EGF-like domain; CRISP: Cysteine-rich secretory protein; Gb: Gigabase; GC: Glutaminyl-peptide cyclotransferase; GO: Gene ontology; HYAL: Hyaluronidase; KUN: Kunitz-type protease inhibitor; LAAO: L amino-acid oxidase; MYO: Myotoxin (crotamine); NGF: Nerve growth factor; NF: Neurotrophic factor; nt: Nucleotide; NUC: Nucleotidase; PDE: Phosphodiesterase; PDI: Protein disulfide isomerase; PLA2: Phospholipase A2; SVMP: Snake venom metalloproteinase (types II and III); SVSP: Snake venom serine proteinase; VEGF: Vascular endothelial growth factor; VESP: Vespryn (ohanin-like); VF: Venom factor; WAP: Waprin.

## Competing interests

The authors declare that they have no competing interests.

## Authors’ contributions

The project was conceived and planned by DR and AL. DR, MM, and KA collected and analyzed the data. DR wrote the manuscript. All authors read and approved the final manuscript.
